# Determination of *ERG*(+), *EZH2*, *NKX3.1*, and *SPINK‐1* subtypes to evaluate their association with clonal origin and disease progression in multifocal prostate cancer

**DOI:** 10.1002/cnr2.1728

**Published:** 2022-10-05

**Authors:** Yenifer Yamile Segura‐Moreno, María Carolina Sanabria‐Salas, Jorge Andrés Mesa‐López De Mesa, Rodolfo Varela‐Ramirez, Natalia Lizeth Acosta‐Vega, Martha Lucía Serrano

**Affiliations:** ^1^ Cancer Biology Research Group Instituto Nacional de Cancerología Bogotá Colombia; ^2^ Department of Chemistry Universidad Nacional de Colombia, Ciudad Universitaria Bogotá Colombia; ^3^ Department of Pathology Instituto Nacional de Cancerología Bogotá Colombia; ^4^ Department of Urology Instituto Nacional de Cancerología Bogotá Colombia; ^5^ Department of Urology Universidad Nacional de Colombia Bogotá Colombia

**Keywords:** clonal evolution, *ERG* gene, *EZH2* gene, genetic heterogeneity, *NKX3.1* gene, prostate cancer, *SPINK‐1* gene, *SPOP* gene, *TMPRSS2‐ERG* fusion

## Abstract

**Background:**

The prognostic relevance of prostate cancer (PCa) molecular subtypes remains controversial, given the presence of multiple foci with the possibility of different subtypes in the same patient.

**Aim:**

To determine the clonal origin of heterogeneity in PCa and its association with disease progression, *SPOP*, *ERG*(+), *EZH2*, *NKX3.1*, and *SPINK‐1* subtypes were analyzed.

**Methods:**

A total of 103 samples from 20 PCa patients were analyzed; foci of adjacent non‐tumor prostate tissue, HGPIN, GL3, GL4, GL5, and LN were examined to determine the presence of the *TMPRSS2‐ERG* fusion and *ERG*, *EZH2*, *NKX3.1*, and *SPINK‐1* expression levels, using RT‐PCR. Mutations in exons 6 and 7 of the *SPOP* gene were determined by sequencing. The presence of subtypes and molecular patterns were identified by combining all subtypes analyzed. To establish the clonal origin of multifocal PCa, molecular concordance between different foci of the same patient was determined. Association of these subtypes with histopathological groups and time to biochemical recurrence (BCR) was assessed.

**Results:**

No mutation was found in *SPOP* in any sample. The *ERG*(+) subtype was the most frequent. The molecular pattern containing all four PCa subtypes was only detected in 3 samples (4%), all LN, but it was the most frequent (40%) in patients. Molecular discordance was the predominant status (55%) when all analyzed molecular characteristics were considered. It was possible to find all subtypes, starting as a preneoplastic lesion, and all but one LN molecular subtype were *ERG*(+) and *NKX3.1* subtypes. Only the expression of the *NKX3.1* gene was significantly different among the histopathological groups. No association was found between BCR time in patients and molecular subtypes or molecular concordance or between clinicopathological characteristics and molecular subtypes of *ERG*, *EZH2*, and *SPINK‐1*.

**Conclusion:**

The predominance of molecular discordance in prostatic foci per patient, which reflects the multifocal origin of PCa foci, highlights the importance of analyzing multiple samples to establish the prognostic and therapeutic relevance of molecular subtypes in a patient. All the subtypes analyzed here are of early onset, starting from preneoplastic lesions. *NKX3.1* gene expression is the only molecular characteristic that shows a progression pattern by sample.

## 1 BACKGROUND

According to the report of the International Agency for Research on Cancer (IARC), prostate cancer (PCa) is the second type of cancer with the highest incidence and the fifth cause of death by cancer worldwide for both sexes and all ages.^1^ PCa starts as a preneoplastic lesion known as high‐grade prostatic intraepithelial neoplasia (HGPIN); it progresses to cancer and can cause regional and distant metastasis. In a large percentage of cases, PCa behaves as an indolent disease, but in other cases, it can act aggressively and cause death. The criteria to establish PCa prognosis include Prostate Specific Antigen (PSA) level, Gleason score, and clinical tumor, node, and metastasis (TNM) stage. Nevertheless, these criteria do not establish a prognosis with sufficient certainty.^2^

Numerous studies have proposed different classifications of PCa molecular subtypes that can contribute to determining prognosis; among them, ETS(+) subtypes characterized by chromosomal fusions that cause the overexpression of ETS transcription factors. The most prevalent ETS fusion in PCa is *TMPRSS2‐ERG*, which leads to *ERG* overexpression.^3^, ^4^, ^5^ *ERG* overexpression, in turn, generates an increase in the expression of *EZH2*, which acts as a histone methyltransferase enzyme, and a decrease in the expression of the *NKX3.1* tumor suppressor gene, which is an androgen‐regulated prostate‐specific homeobox gene and a transcription factor. *NKX3.1* also binds to *TMPRSS2* upstream sequences, and in *TMPRSS2‐ERG* cases, it negatively regulates *ERG* expression and is a negative feedback control since *ERG* directly represses *NKX3.1*.^6^, ^7^ *EZH2* and *NKX3.1* had also been described as PCa subtypes. Another subtype reported in PCa is characterized by *SPINK‐1* overexpression, which specifically happens in a subset of ETS(−) and is associated with another subtype, the *SPOP* subtype, characterized by a mutation in this gene.^3^ *SPINK‐1* protein is a trypsin inhibitor, which can function as an autocrine growth factor,^8^, ^9^ while *SPOP* is an E3 ubiquitin ligase substrate‐binding subunit of the proteasome complex that mediates the ubiquitination of target proteins, leading more frequently to their proteasomal degradation.^10^

The prognostic relevance of PCa molecular subtypes remains controversial.^11^, ^12^, ^13^, ^14^, ^15^, ^16^, ^17^, ^18^, ^19^, ^20^ The presence of multiple foci with the possibility of different subtypes in the same patient with PCa could contribute to the difficulty of predicting the clinical behavior of a specific PCa subtype.^21^, ^22^, ^23^ It is not well known whether these foci have a monoclonal origin that expands through the prostate, giving rise to new foci that share some molecular characteristics by origin but begin to acquire changes in subclonal events; or whether each focus appears independently, having a multiclonal origin, so they are expected to be very heterogeneous at the molecular level.^22^, ^24^, ^25^

To elucidate the origin of heterogeneity in PCa and its association with disease progression, *ERG*(+), *SPOP*, *EZH2*, *SPINK‐1*, and *NKX3.1* subtypes were analyzed in 103 samples from prostatic foci and regional metastasis to lymph nodes (LN) from 20 PCa patients with poor prognosis. Molecular patterns are also established by sample and patient, combining the presence of all subtypes analyzed.

## 2 METHODS

### 2.1 Samples

Formalin‐fixed paraffin‐embedded (FFPE) tissue samples obtained from radical prostatectomy (RP) of 20 PCa patients with poor prognosis (regional metastases and/or biochemical recurrence) and 5‐year follow‐up were included. Cases were chosen from the project “Exploration of potential predictor biomarkers in patients diagnosed with prostate cancer,” approved by the Ethics Committee of the Instituto Nacional de Cancerología (Bogotá, Colombia) to develop this study. Since tissues were obtained as part of routine care and not specifically for this study, and the data used in this study were previously anonymized, the Ethics Committee of the Instituto Nacional de Cancerología (Bogotá, Colombia) waived the need for informed consent for this study.

Four to five FFPE samples per patient were selected to identify foci with different degrees of differentiation; a total of 83 samples were analyzed. These samples corresponded to HGPIN (*n =* 17), PCa foci with different Gleason scores (*n =* 53; GL3 *n =* 23; GL4 *n =* 23; and GL5 *n =* 7), and LN (*n =* 13). A sample of adjacent non‐tumor prostate tissue was used from each patient as a reference (*n =* 20). Two frozen PCa tissue samples were also used as a positive control of the *TMPRSS2‐ERG* fusion, as well as the PC3 cell line was assessed as a negative control for gene fusions. All methods complied with relevant guidelines and regulations at the national and international levels.

### 2.2 Cell culture

The PC3 cell line was derived from androgen‐independent PCa. This line was kindly provided by Dr. Fabio Aristizabal, from the Universidad Nacional de Colombia. The culture conditions were as follows: 5% CO_2_ at 37°C, in DMEM medium (Dulbecco/Vogt modified Eagle's minimal essential medium), supplemented with 10% fetal bovine serum and a cocktail of penicillin, streptomycin, and 1% amphotericin B.

### 2.3 DNA/RNA extraction and quantification from FFPE tissue samples

DNA/RNA was isolated from FFPE tissue by automated extraction in the QIAcube using a modified protocol of the commercial kit AllPrep DNA/RNA FFPE (QIAGEN, Hilden, Germany). The modification consisted of incubating the sample before starting the automated protocol with proteinase K at 56°C for 3 h, instead of 15 min. The RNA was quantified by using the Qubit® 2.0 fluorometer RNA HS assay (Invitrogen, Waltham, MA). The RNA was stored at −70°C until its use. Good quality DNA was obtained with this same kit, which was also quantified using the Qubit® 2.0 fluorometer DNA HS assay and stored at −20°C.

### 2.4 Analysis of the presence or absence of the *TMPRSS2‐ERG* fusion

RT‐PCR was performed to determine the presence of the *TMPRSS2‐ERG* fusion (exon 1 of *TMPRSS2*: exon 4 of *ERG*), using the KAPA SYBR FAST One‐Step kit to carry out qRT‐PCR (Kapa Biosystems, Wilmington, MA), following the recommendations of the manufacturer. The primers used are presented in Table 1.^5^ It was run in the PTC‐200 Thermal Cycler (PCR System) (MJ Research, Reno, NV). The protocol begins with reverse transcription at 42°C for 5 min for 1 cycle; activation of reverse transcriptase at 95°C for 3 min for 1 cycle; denaturation of samples at 95°C for 10 s, annealing at 61°C for 20 s, and extension at 72°C for 1 s for 45 cycles. The presence or absence of the *TMPRSS2‐ERG* fusion was determined by visualizing the corresponding amplimers in 2% agarose gels with SYBR Safe.

**TABLE 1:** Primers used in the study for RT‐PCR, qRT‐PCR, and PCR assays with their respective standardized optimal conditions.

Gene	Primers	Amplicon length (bp)	Annealing temperature (°C)	Primer concentration (nM)	References
*TMPRSS2‐ERG* (exon 1: exon 4)	F: TAGGCGCGAGCTAAGCAGGAG	125	61	100	5
	R: GTAGGCACACTCAAACAACGACTGG				
*SPOP* ‐6	F: ACCCATAGCTTTGGTTTCTTCTCCC	170	55	200	26
	R: TATCTGTTTTGGACAGGTGTTTGCG				
*SPOP* ‐7	F: ACTCATCAGATCTGGGAACTGC	240	55	200	26
	R: AGTTGTGGCTTTGATCTGGTT				
*ERG*	F: TCTTGGACCAACAAGTAGCC	151	63	150	27
	R: GTCGGGATCCGTCATCTTG				
*EZH2*	F: TGTGGATACTCCTCCAAGGAA	90	55	250	28
	R: GAGGAGCCGTCCTTTTTCA				
*NKX3.1*	F: CCGAGACGCTGGCAGAGA	81	55	200	29
	R: GAAGGGCGCCTGAAGTGTT				
*SPINK‐1*	F: CAAAAATCTGGGCCTTGCTGAGAAC	62	55	200	8
	R: AGGCCTCGCGGTGACCTGAT				
*UBC*	F: ATTTGGGTCGCGGTTCTTG	133	55	150	30
	R: TGCCTTGACATTCTCGATGGT				

*Note*: F, forward; R, reverse.

### 2.5 Analysis of the presence or absence of *SPOP* mutations

*SPOP* mutations were identified by sequencing exons 6 and 7, which were amplified through PCR with the Platinum High Fidelity PCR kit (Invitrogen, Waltham, MA), using the SPOP‐6 and SPOP‐7 primers^26^ (Table 1), with the following cycling conditions: incubation at 94°C for 2 min, denaturation at 94°C for 30 s, primer annealing at 55°C for 30 s, and extension at 68°C for 1 min; 35 cycles were run. Subsequently, for sequencing, the BigDye Terminator v3.1 Cycle Sequencing Kit (Applied Biosystem, Foster City, CA) was used with the same PCR primers, following the manufacturer's recommendations. The sequences were purified according to the BigDye XTerminator KIT protocol (Applied Biosystem, Foster City, CA), and obtained using the 3500 Genetic Analyzer (Applied Biosystem, Foster City, CA).

### 2.6 RT‐PCR for *ERG* , *EZH2* , *NKX3.1*, *SPINK‐1*, and *UBC* gene expression

RT‐PCR was carried out in a single step using the qScript 1‐Step SYBR Green qRT‐PCR kit (Quanta Biosciences, Cummings Center, Beverly, MA), following the manufacturer's instructions. The protocol begins with reverse transcription at 50°C for 10 min for 1 cycle; activation of reverse transcriptase at 95°C for 5 min for 1 cycle; denaturation of samples at 95°C for 10 s, annealing for 20 s, and extension at 72°C for 1 min for 45 cycles. The primers used for each gene and respective amplicon length, concentration, and annealing temperature are described in Table 1.^8^, ^27^, ^28^, ^29^, ^30^ A LightCycler® 480 thermal cycler (Roche Life Science, Penzb*ERG*, Upper Bavaria, Germany) was used for RT‐qPCR. To obtain the melting curves, the PCR products were heated at 95°C for 5 s, at 65°C for 1 min, at 97°C continuously with 5–10 adq/°C, and then cooled at 40°C for 10 s. *UBC* was used as a reference expression gene. All tests were carried out in duplicate. All 103 study samples showed similar values of *UBC* gene expression: cross points (CPs) median = 29.61, mean = 29.68.

### 2.7 Elaboration of *ERG* , *EZH2* , *NKX3.1*, *SPINK‐1*, and *UBC* gene expression standard curves

The standard curves were prepared with PCR products obtained from a FFPE sample of adjacent non‐tumor prostate tissue, purified from agarose gel using the Illustra GFX PCR DNA and Gel Band Purification commercial kit (GE Healthcare, Little Chalfont, United Kingdom), and quantified using the Qubit® 2.0 fluorometer DNA HS assay (Invitrogen, Waltham, MA). The number of copies of the cDNA was calculated using the URI Genomics & Sequencing Center's online calculator (2004) (http://cels.uri.edu/gsc/cndna.html). Seven serial dilutions were made in duplicate, between 1 × 10^1^ and 1 × 10^7^ copy number. The efficiency of the amplification (E) was calculated as: *E* = 10[−1/slope]. *E* values were: 1.903 for UBC, 1.873 for *EZH2*, 2.003 for *NKX3.1*, and 1.904 for *SPINK‐1*; *SD*s were <0.2 with respect to the duplicates. The validity of the points, efficiency calculations, and errors in the equipment were verified by the method of the second derivative.

### 2.8 Relative expression of *ERG* , *EZH2* , *NKX3.1*, and *SPINK‐1*

With the E values from the standard curves, the relative expression of each sample was calculated using the method of Pfaffl,^31^ with the following equation:

$$\text{Relative expression}=\left[\frac{{E_{\mathrm{ref}}}^{{\mathrm{CP}}_{\mathrm{ref}}}\;\text{Sample}}{{E_{\text{targ}}}^{{\mathrm{CP}}_{\text{targ}}}\;\text{Sample}}\right]÷\frac{{E_{\mathrm{ref}}}^{{\mathrm{CP}}_{\mathrm{ref}}}\;\text{Calibrator}}{{E_{\text{targ}}}^{{\mathrm{CP}}_{\text{targ}}}\;\text{Calibrator}},$$

where *E*_target_ is the E of the target gene; *E*_ref_ is the E of the reference gene (UBC); CP_target_ is the cross point of the target gene; *CP*_ref_ is the CP of the reference gene (UBC). Calibrator is the adjacent non‐tumor prostate tissue for each patient. To analyze the relative expression of *ERG*, *EZH2*, *NKX3.1*, and *SPINK‐1*, the values obtained were converted into three categories: highly expressed (>1.20), normally expressed (between 0.80 and 1.20), and lowly expressed (<0.80).

### 2.9 Determination of PCa molecular subtypes

PCa molecular subtypes were assigned as follows: *ERG*(+) subtype, when *TMPRSS2‐ERG* fusion is present and/or *ERG* mRNA is highly expressed; *SPOP* subtype, in the presence of mutations in the *SPOP* gene; *EZH2* subtype, when *EZH2* mRNA is highly expressed; *NKX3.1* subtype, when *NKX3.1* mRNA is lowly expressed; and *SPINK‐1* subtype, when *SPINK‐1* mRNA is highly expressed.

These subtypes were assigned by sample and patient; to consider that a patient is positive for a subtype, at least one sample must have this subtype. A molecular pattern is the combination of the presence of all subtypes analyzed in samples and patients.

### 2.10 Concordance patterns of the presence/absence of the *TMPRSS2‐ERG* fusion, *SPOP* mutations, and gene expression of *ERG* , *EZH2* , *NKX3.1*, and *SPINK‐1* in prostatic foci of patients

**FIGURE 1:**
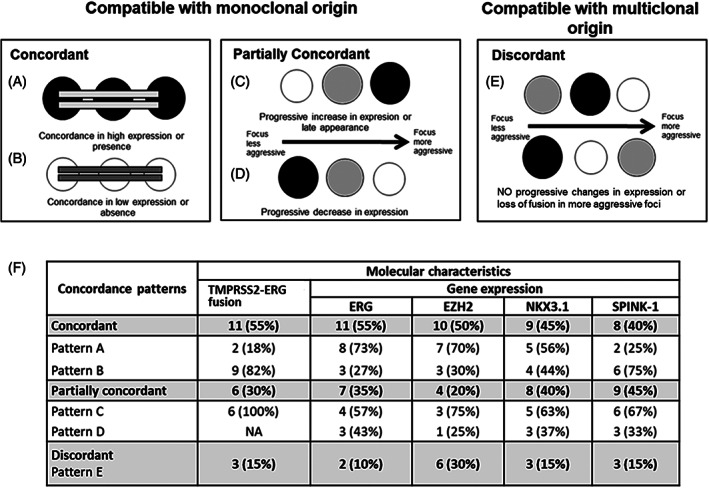
Concordance patterns found for each molecular characteristic analyzed among prostatic foci of each patient. Color pattern of gene expression or fusion: black: highly expressed (>1.20) or presence of fusion; medium gray: normally expressed (0.80–1.20); white: lowly expressed (<0.80). There are five concordance patterns (Figure 1A–E). Two concordant patterns (A, B): (A) Homogeneous agreement of high expression or presence of fusion; (B) Homogeneous agreement of low expression or absence of fusion. Two partially concordant patterns (C, D): (C) Progressive increase in gene expression or late appearance of fusion related to tumor progression; (D) Progressive decrease in expression related to tumor progression. One discordant pattern (E): (E) Molecular mismatch among different foci independently of lesion progression, or disappearance of fusion with tumor progression since once the fusion appears, it is expected not to disappear. Concordant and partially concordant patterns are compatible with monoclonal origin. The discordant pattern is compatible with multiclonal origin. (F) Concordance patterns with respect to each molecular alteration evaluated by patient.

Concordance for the presence/absence of the *TMPRSS2‐ERG* fusion, *SPOP* mutations, and *ERG*, *EZH2*, *NKX3.1*, and *SPINK‐1* gene expression was assessed separately and together, aiming to establish the clonal origin of all prostatic foci (HGPIN and PCa) analyzed by patient. LN were not used for concordance analyses, given that they are extraprostatic lesions. Figure 1A–E graphically describe the five concordance patterns found: two concordant patterns (patterns A and B), two partially concordant patterns (patterns C and D), and one discordant pattern (pattern E).

### 2.11 Data analysis

Clinicopathological characteristics were analyzed as continuous variables: age at diagnosis (years), prediagnosis PSA (ng/ml), tumor percentage in RP, index diameter (cm), and time to BCR (months), as well as categorical variables: Gleason score, Gleason grade group at RP, margin status in RP, perineural invasion, lymphovascular invasion, primary tumor size and/or amount of spread into nearby structures (pT), lymph node compromise (pN), and 5‐year BCR. Molecular characteristics were analyzed as categorical variables: detection of the *TMPRSS2‐ERG* fusion, *SPOP* mutations, and relative expression of *ERG*, *EZH2*, *NKX3.1*, and *SPINK‐1*. The values obtained were converted into three categories: highly expressed (>1.20), normally expressed (between 0.80 and 1.20), and lowly expressed (<0.80). The distribution of the relative expression of *ERG*, *EZH2*, *NKX3.1*, and *SPINK‐1* was also analyzed.

The assumption of normality in the quantitative variables was validated using the Shapiro–Wilk test. For the variables that fulfilled the assumption of normality, the mean (*SD*) was used, while the median (interquartile range) was employed for those that did not. For comparison between groups, Student's t test for independent samples was applied in the case of variables with normal distribution, and the Wilcoxon signed‐rank test otherwise. In the case of categorical variables, absolute and relative frequencies were obtained as descriptive measures. Given the sample size, the Fisher exact test was applied to determine whether there was a statistically significant association between the categorical variables.

The Shapiro–Wilk test showed that relative gene expression data did not comply with the normality criteria in any histopathological group. Although the data complied with normality when using log2 transformation, they did not fulfill the assumption of homogeneity of variances according to Levene's test. Significant differences were verified by groups with the molecular alterations evaluated in the study, using the Kruskal‐Wallis nonparametric test for categorical variables and the Fisher test for quantitative variables. According to the results obtained with the Kruskal‐Wallis test, a paired comparison was subsequently applied between histopathological groups and clinico‐histopathological characteristics. The results were considered significant with a *p* < .05, using the Mann–Whitney U‐test, and visualized according to the distribution of median values and data dispersion, plotted on a logarithmic scale log2 with a box‐and‐whisker diagram. All analyses were carried out with the statistical software SPSS v18.

## 3 RESULTS

### 3.1 Clinicopathological characteristics of the cohort

A total of 103 samples from 20 PCa patients were analyzed: foci of adjacent non‐tumor prostate tissue (*n =* 20), HGPIN (*n =* 17), GL3 (*n =* 23), GL4 (*n =* 23), GL5 (*n =* 7), and LN (*n =* 13). The main clinicopathological characteristics of the 20 patients are summarized in Table 2.

**TABLE 2:** Clinicopathological and molecular characteristics of the full cohort, classified by BCR time, categorized as ≤3 and >3 months

Characteristics	Full cohort (*n =* 20)	BCR time^a^		*p*‐value
		≤3 months (*n =* 9)	>3 months (*n =* 8)	
Age at diagnosis (years)				
Median [IQR]	66.0 [12.5]	65.0 [12.0]	63.0 [21.5]	1.00
Prediagnosis_PSA				
Median [IQR]	14.3 [12.7]	14.3 [9.75]	11.5 [11.8]	0.681
Gleason score, *n* (%)				
7	19	8 (50.0)	8 (50.0)	1.00
8	1	1 (100)	0 (0.00)	
Gleason grade group at RP (%)				
G2	5	2 (40.0)	3 (60.0)	1.00
G3	14	6 (54.5)	5 (45.5)	
G4	1	1 (100)	0 (0.00)	
Tumor percentage (%)				
Mean ± *SD*	39.3 ± 19.2	49.4 ± 21.6	29.9 ± 12.5	0.040^b^
Index diameter				
Mean ± *SD*	2.00 ± 0.48	2.19 ± 0.55	1.79 ± 0.39	0.110
Margin status in RP, *n* (%)				
Negative	4	1 (33.3)	2 (66.4)	0.576
Positive	16	8 (57.2)	6 (42.8)	
Perineural invasion, *n* (%)				
Intraprostatic	8	3 (50.0)	3 (50.0)	1.00
Intraprostatic + extraprostatic	12	6 (54.5)	5 (45.5)	
Lymphovascular invasion, *n* (%)				
No	10	2 (25.0)	6 (75.0)	0.131
Yes	9	6 (75.0)	2 (25.0)	
Unknown	1	1 (100)	0 (0.00)	
Pathologic stage. pT, *n* (%)				
2c‐3a	7	1 (16.7)	5 (83.3)	0.049^b^
3b	13	8 (72.7)	3 (27.3)	
Pathologic stage. pN, *n* (%)				
0	4	0 (0.00)	4 (100)	0.029^b^
1	16	9 (69.2)	4 (30.8)	
Fusion, *n* (%)				
Positive	10	4 (50.0)	4 (50.0)	1.00
Negative	10	5 (55.6)	4 (44.4)	
ERG, *n* (%)				
Positive	14	6 (54.6)	5 (45.4)	1.00
Negative	6	3 (50.0)	3 (50.0)	
EZH2, *n* (%)				
Positive	15	5 (41.7)	7 (58.3)	0.294
Negative	5	4 (80.0)	1 (20.0)	
NKX3.1, *n* (%)				
Positive	9	4 (57.2)	3 (42.8)	1.00
Negative	11	5 (50.0)	5 (50.0)	
SPINK‐1, *n* (%)				
Positive	12	4 (36.4)	7 (63.6)	0.131
Negative	8	5 (83.3)	1 (16.7)	
Concordance pattern, *n* (%)				
Concordant	3	2 (66.7)	1 (33.3)	1.00
Partially concordant	6	2 (40.0)	3 (60.0)	
Discordant	11	5 (55.6)	4 (44.4)	

Abbreviations: IQR, interquartile range; RP, radical prostatectomy.

^a^ In 3 cases out of 20, it was not possible to establish BCR.

^b^ <0.05.

### 3.2 Determination of molecular subtypes and molecular patterns by sample and patient

The molecular subtypes analyzed in this study were: *ERG*(+), *EZH2*, *NKX3.1*, and *SPINK‐1*. The *ERG*(+) subtype includes the presence of the *TMPRSS2‐ERG* fusion and/or highly expressed *ERG* mRNA. Since no mutations were found in the *SPOP* gene, this was not considered for analysis. A total of 83 samples (70 prostatic foci and 13 LN) obtained from 20 patients were analyzed. All the results by patient and sample are shown in Table 3.

**TABLE 3:** Determination of molecular characteristics, subtypes, and molecular patterns by focus and patient, presented by concordance pattern of all molecular characteristics in prostatic foci by patient.

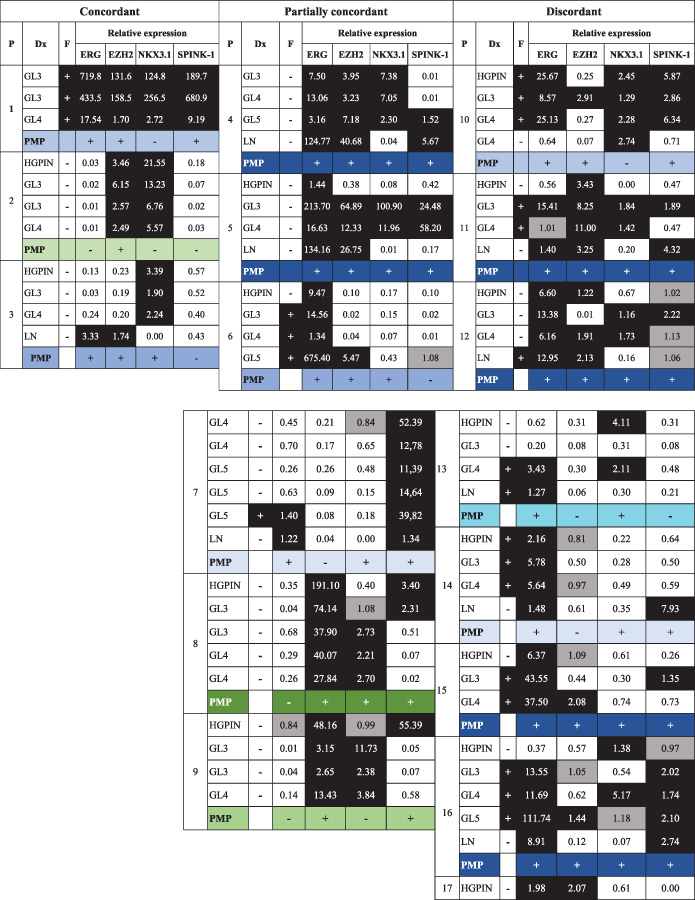
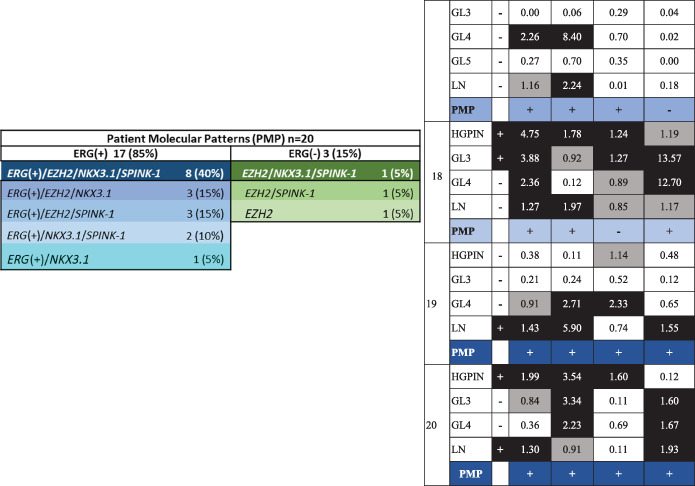

*Note*: Color pattern of gene expression or fusion: *black*: highly expressed (>1.20) or presence of fusion; medium gray: normally expressed (0.80–1.20); *white*: lowly expressed (<0.80). The lower small table shows patient molecular patterns (PMP). These PMP were assigned considering whether any of the samples analyzed (prostatic or node samples) presented (+) the molecular subtype that favored disease progression (ERG[+], EZH2 high, NKX3.1 low, SPINK‐1 high) or not (−). Fusion was not considered for PMP because high ERG includes it. Color pattern of PMP: Each color represents a different pattern. Eight different patterns were found: five ERG(+) (blue) and three ERG(−) (*green*). The most frequent patterns were ERG(+) in 17 patients (85%) and the most frequent pattern was the darkest *blue* pattern: ERG(+)/EZH2/NKX3.1/SPINK‐1 (in 8 patients). ERG(−) patterns were found in 3 patients (15%) and are colored in *green*; all of these are different.

Abbreviations: Dx, histopathological diagnosis; F, TMPRSS2‐ERG fusion; GL, Gleason pattern; HGPIN, high‐grade prostatic intraepithelial neoplasia (preneoplastic lesion); LN, lymph node (regional metastasis); P, patient.

**FIGURE 2:**
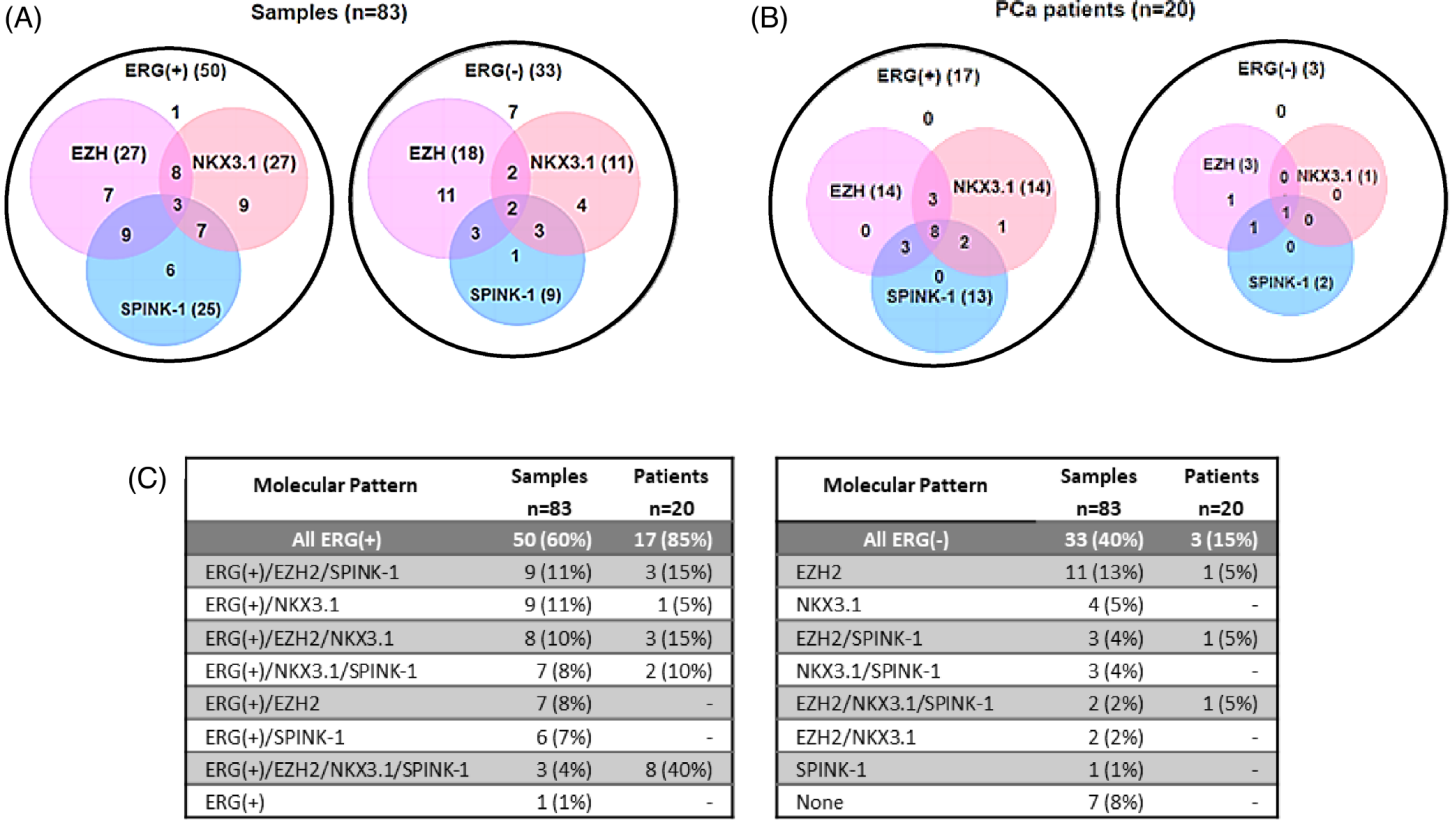
Molecular subtypes and molecular patterns by sample and patient. (A) Venn diagrams showing the presence of different molecular patterns by sample. Numbers between parentheses correspond to the number of samples with a determined specific subtype, and the number without parentheses is for each molecular pattern. (B) Venn diagrams showing the presence of different subtypes and molecular patterns by patient. Numbers between parentheses correspond to the number of patients with a specific subtype, and the number without parentheses is for each patient's molecular pattern. (C) Frequency table of molecular patterns by sample and patient.

Figure 2A shows the presence of different molecular subtypes by samples and divided by the presence/absence of the *ERG*(+) subtype, which is considered the most frequent and most widely studied finding in PCa. The presence of each subtype in the samples analyzed ranges from 41% to 60%; 50 samples (60%) were considered *ERG*(+), 45 (54%) were *EZH2*, 38 (46%) were *NKX3.1*, and 34 (41%) were *SPINK‐1*. A nonmolecular subtype was identified in 7 samples. The presence of each subtype in the samples was predominantly in combination with *ERG*(+), ranging from 60% to 74%: 27 (60%) of *EZH2* samples, 27 (71%) of *NKX3.1* samples, and 25 (74%) of *SPINK‐1* samples. *ERG*(+) alone was found in only 1 sample (Figure 2A).

To establish patient subtypes, a patient is considered positive for a subtype when they have at least one sample with the subtype present. The presence of each subtype in the samples analyzed ranges from 75% to 85%; there were 17 patients (85%) with *ERG*(+), 17 (85%) with *EZH2*, 15 (75%) with *NKX3.1*, and 15 (75%) with *SPINK‐1*. The presence of each subtype in patients was predominantly in combination with *ERG*(+), ranging from 82 to 93%; *ERG*(+) alone was not found in any patient (Figure 2B).

When considering the four subtypes simultaneously, 15 molecular patterns were identified in the samples, with the frequency of these patterns ranging from 1% to 13%; 8 molecular patterns were *ERG*(+), and they were found in 50 samples (60%), while 7 were *ERG*(−) in 33 samples (40%) (Figure 2A,C). The simultaneous presence of *ERG*(+)/*EZH2*/*NKX3.1*, which are related among them, was found in 11 samples (13%): 8 samples (9.6%) with this pattern and 3 samples (3.6%) also including *SPINK‐1* (*ERG*(+)/*EZH2*/*NKX3.1*/*SPINK‐1*). The most frequent pattern was *EZH2* alone (13%), followed by *ERG*(+)/*EZH2*/*SPINK‐1* and *ERG*(+)/*NKX3.1* (11% each).

A total of 8 different molecular patterns were found in patients, with a frequency ranging from 5% to 40%; 5 molecular patterns were *ERG*(+), and they were found in 17 patients (85%), while 3 patterns were *ERG*(−) in 3 patients (15%) (Figure 2B,C). The simultaneous presence of *ERG*(+)/*EZH2*/*NKX3.1*, which are related among them, was found in 11 patients (55%): 3 samples (15%) with this pattern and 8 samples (40%) also including *SPINK‐1* (*ERG*(+)/*EZH2*/*NKX3.1*/*SPINK‐1*). The latter was the most frequent pattern in patients. The 3 molecular patterns with *ERG*(−) are present in only 1 patient each (5%), but all have *EZH2* subtype (Figure 2B,C).

### 3.3 Determination of concordance for each molecular alteration evaluated in prostatic foci

Aiming to establish the clonal origin of the different prostatic foci analyzed in each of the 20 patients, the concordance of molecular characteristics was assessed with respect to the presence or absence of the *TMPRSS2‐ERG* fusion and the *ERG*, *EZH2*, *NKX3.1*, and *SPINK‐1* gene expression in 70 prostatic foci (17 HGPIN and 53 PCa) (Figure 1, Table 3). LN samples were excluded from these analyses.

Figure 1A–E show five possible concordance patterns found: two concordant (figures A,B), two partially concordant (figures C,D), and one discordant (figure E). The concordant and partially concordant patterns are compatible with monoclonal origin because discordances related to lesion progression findings in the partially concordant pattern are considered subclonal events; one discordant pattern is compatible with multiclonal origin. Figure 1F shows concordance patterns with respect to each molecular alteration evaluated by patient. The concordant pattern was the predominant state for all evaluated alterations (40 to 55%), except for *SPINK‐1* expression, where the partially concordant state was the predominant pattern in 45%. The partially concordant pattern ranged between 20% and 45%, and total molecular concordance, which includes concordant and partially concordant cases, was the most common pattern for all molecular characteristics analyzed, ranging between 70% and 90% (90% for *ERG*, 85% for fusion status, *NKX3.1* and *SPINK‐1* expression, and 70% for *EZH2* expression), which is compatible with monoclonal origin. Discordance was the least common pattern (10%–30%), which would support a multiclonal origin (Figure 1F).

Among the cases with a concordant pattern, the most common was overexpression of *ERG* (73%), *EZH2* (70%), and *NKX3.1* (56%) (pattern A), as well as the absence of fusion (82%) and underexpression of *SPINK‐1* (75%) (pattern B). The most common pattern for partially concordant cases was pattern C: acquisition of the fusion (100%) and progressive increase in gene expression of *ERG* (57%), *EZH2* (75%), *NKX3.1* (63%), and *SPINK‐1* (67%) (Figure 1F).

### 3.4 Determination of concordance in prostatic foci for all molecular alterations evaluated in each patient

After analyzing concordance for each molecular characteristic separately, concordance was evaluated simultaneously for all molecular characteristics analyzed in this study in prostatic foci (*n =* 70) (Table 3). A total of 3 out of 20 patients were concordant (15%), 6 patients were partially concordant (30%), and 11 patients were discordant (55%), which shows that heterogeneity and the possibility of multiclonal origin increases from 15 to 30% by molecular characteristic analyzed separately to 55% when the five molecular characteristics are analyzed simultaneously.

Intratumoral concordance was also assessed in foci with the same GL score in 6 patients who had samples with more than one focus with the same GL. Although the foci of the same GL in patients 1, 2, and 9 showed concordance, there were differences in patients 7, 10, and 8 in the presence of fusion and expression of *ERG*, *NKX3.1*, and *SPINK‐1*, which indicates intratumoral heterogeneity (Table 3).

### 3.5 Molecular alterations in metastasis concerning prostatic foci

Metastatic material from the lymph nodes of 13 patients was analyzed (Table 3). None of the LN analyzed showed the same pattern as any of the other foci examined in the prostate of each patient, which is the expected outcome due to the subclonal events suffered by the cells to metastasize. To establish the possible origin of the metastases found in the LN of the 13 cases, when this material was available, the molecular patterns of the LN and prostatic lesions were compared to identify how the lesion could have caused these metastases to LN.

Table 4 shows the differences found between LN and lesion(s) most similar to LN in each of the 13 cases. Noticeably, in most cases—6 out of 13 (46.2%)—the LN differs from the most similar focus by a single molecular characteristic. The table shows that in 2 of the 6 cases that behave this way, two different foci have the same molecular pattern (cases 7 and 17). In 4 of the 13 cases analyzed (30.7%), two differences were found; in 2 cases (cases 11 and 18), two foci with a different pattern had the same level of similarity with the corresponding LN. As for the 2 cases that presented three differences in this type of comparison (cases 3 and 20), they both showed the same number of differences in all prostate foci examined, which indicates that it is impossible to identify correspondence with a particular focus. A single case presented four differences regarding the PCa foci with different patterns in each (case 19).

**TABLE 4:** Matching lymph node and localized prostatic lesions to show which neoplastic foci contributed to lymph node metastasis.

Case	Most similar prostate lesion	Observed change in LN with respect to the most similar prostate lesion					Total number of references (% of cases)
		Fusion	ERG	EZH2	NKX3.1	SPINK‐1	
4	GL5	—	—	—	↓	—	1 (46.2%)
5	HGPIN	—	—	↑	—	—	
7	GL4 and GL5	—	↑	—	—	—	
12	HGPIN	Gain	—	—	—	—	
13	GL4	—	—	—	↓	—	
17	HGPIN and GL4	—	↓	—	—	—	
11	HGPIN	—	↑	—	—	↑	2 (30.7%)
	GL3	Loss	—	—	↓	—	
14	GL3	Loss	—	—	—	↑	
16	GL4	Loss	—	—	↓	—	
18	HGPIN	Loss	—	—	↓	—	
	GL4	—	—	↑	—	↓	
3	All: HGPIN, PCa	—	↑	↑	↓	—	3 (15.4%)
20	HGPIN	—	—	↓	↓	↑	
	GL3 and GL4	Gain	↑	↓	—	—	
19	GL3	Gain	↑	↑	—	↑	4 (7.7%)
	GL4	Gain	↑	—	↓	↑	
Total		7 cases (3 with gain, 4 with loss)	6 cases (5 ↑, 1 ↓)	5 cases (4 ↑, 1 ↓)	8 cases (8 ↓)	5 cases (4 ↑, 1 ↓)	

*Note*: ↑, increase; ↓, decrease; –, no change.

In their great majority, the differences found are those associated with progression, increase in the expression of ERG, EZH2, SPINK‐1, and decrease of NKX3.1. As for fusion, it is more common to lose it than gain it, which initially would not be expected; however, in the 4 cases with loss of fusion (cases 11, 14, 16, and 18), the ERG expression is not decreased.

### 3.6 Histopathological and clinical significance of the presence of *ERG* , *EZH2* , *SPINK‐1*, and *NKX3.1* subtypes

**FIGURE 3:**
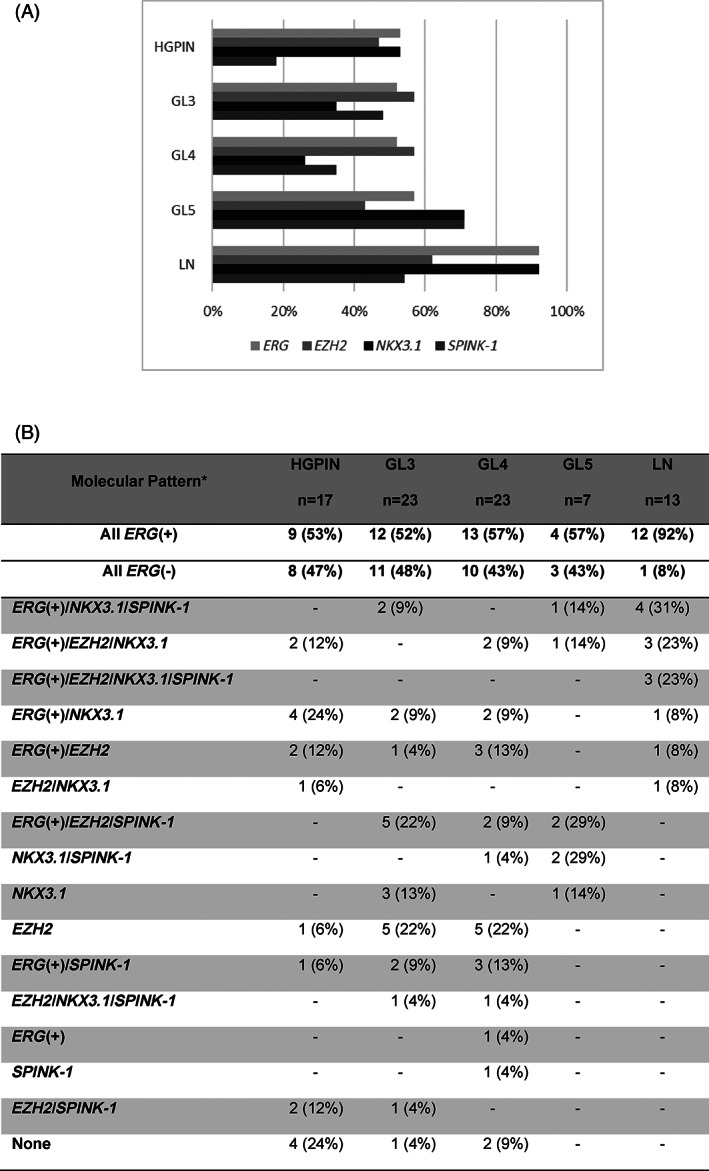
Molecular subtypes and molecular patterns analyzed by histopathological group. (A) Frequency of PCa molecular subtypes. (B) Frequency of molecular patterns by histopathological group. “*” The molecular patterns were organized so that they allow showing less heterogeneity in more advanced groups (GL5 and LN).

Figure 3A shows the frequency of molecular subtypes by histopathological group. All analyzed subtypes were detected starting from the preneoplastic lesion (HGPIN) in a range of 47%–53%, but the *SPINK‐1* subtype had a low percentage (18%). In LN, the range was between 54% and 92%; the presence of each subtype increased compared to HGPIN, where the most frequent subtypes were *ERG* and *NKX3.1* (92% each). It is clear that *ERG* overexpression increases in frequency from HGPIN to LN (53%–92%).

Figure 3B shows the frequency of 15 molecular patterns by histopathological group. A noteworthy finding was that pattern heterogeneity decreased with lesion progression; while 7 molecular patterns were found in HGPIN, 9 in GL3, and 11 in GL4, only 5 were found in GL5 and GL6 in LN. All LN and GL5 have a molecular pattern, but in 7 foci—4 HGPIN (24%), 1 GL3 (4%), and 2 GL4 (9%)—no pattern was found, which probably reflects different patterns not identified with the molecular subtypes analyzed here and increased heterogeneity in these histopathological groups. The molecular pattern with the 4 PCa subtypes (*ERG*(+)/*EZH2*/*NKX3.1*/*SPINK‐1*) was detected only in LN, in 23% of them. The constant in all but one LN molecular pattern was the *NKX3.1* subtype.

**FIGURE 4:**
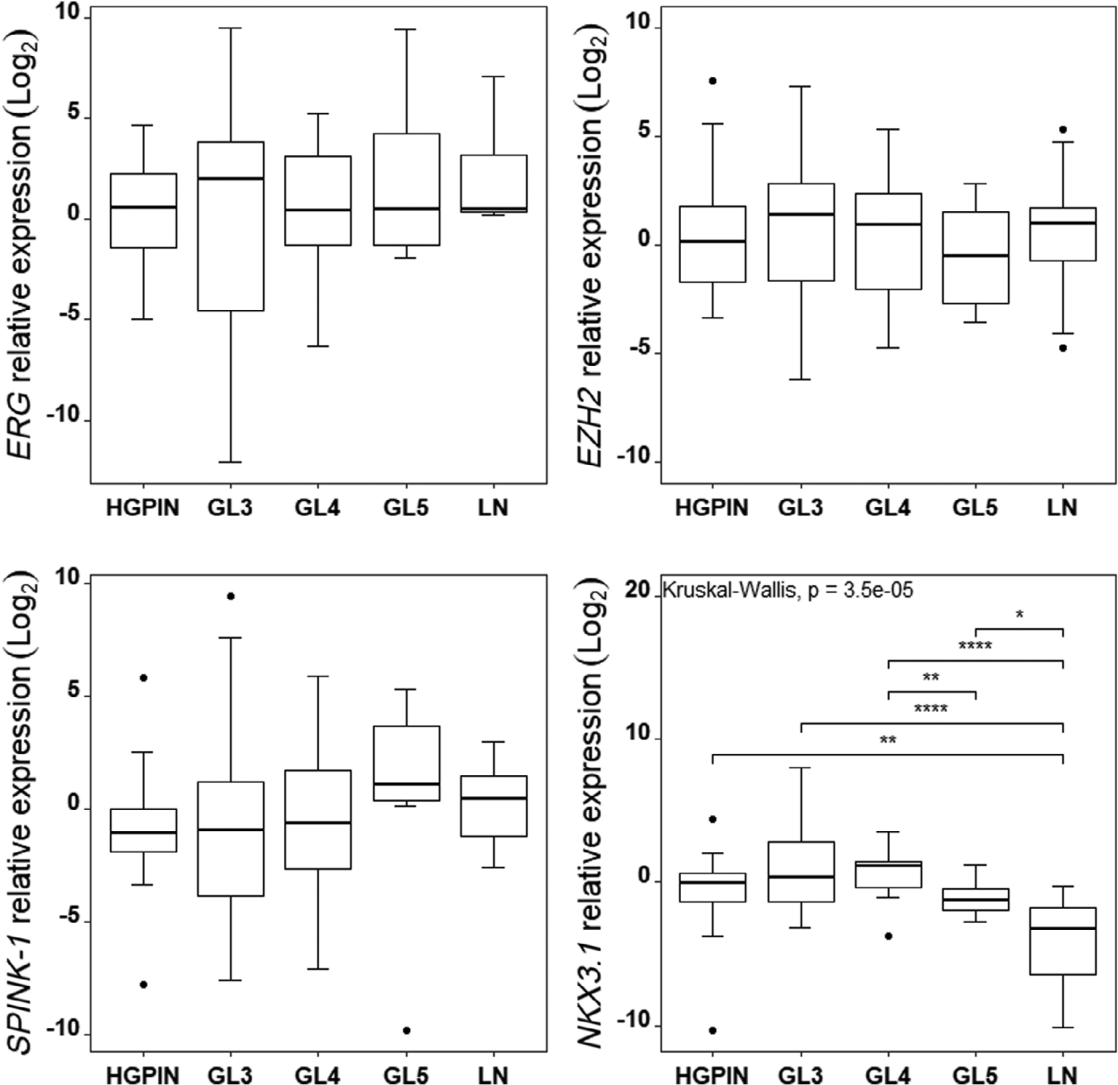
Distribution of the relative expression of *ERG*, *EZH2*, *SPINK‐1*, and *NKX3.1* by histopathological group. Data were graphed using a logarithmic scale (log2). The points are outliers. The Kruskal‐Wallis nonparametric test for the log2 (*NKX3.1*) for the four groups shows a statistically significant difference (*p* = 3.5e‐05). Braces with asterisk represent significant differences (*p <0 .05; **p < 0.01; ***p < 0.001) between the groups, compared with the Mann–Whitney U test statistic.

Association between relative gene expression of *ERG*, *EZH2*, *SPINK‐1*, and *NKX3.1* and histopathological group was also evaluated. The distribution of the relative gene expression of *ERG*, *EZH2*, and *SPINK‐1* showed no significant differences between histopathological groups, but the relative gene expression of *NKX3.1* showed a statistically significant difference between groups (*p* < 0.001). A lower expression in LN samples with respect to all the other groups (LN vs. HGPIN, *p* = 0.0015; LN vs. GL3, *p* < 0.001, LN vs. GL4, *p* < 0.001; LN vs. GL5, *p* = 0.0236), and between GL5 compared to GL4 samples (*p* = .0071), indicates that there is an inversely proportional association between PCa progression (HGPIN, GL3, GL4 vs. GL5, LN) and the expression levels of *NKX3.1* (Figure 4).

An analysis was carried out by comparing clinicopathological characteristics, molecular subtypes, and concordance patterns in all 20 patients by BCR time, categorized as ≤3 and >3 months (Table 2). A statistically significant association was evidenced between BCR time with tumoral percentage (*p* = 0.040), pT (*p* = 0.049), and pN (*p* = 0.029), but no association was found between BCR time and molecular subtypes or molecular concordance. A comparison of the clinicopathological characteristics by molecular subtype with *ERG*, *EZH2*, *NKX3.1*, and *SPINK‐1* subtypes did not show any statistically significant association (Tables S1–S4).

## 4 DISCUSSION

Although PCa is a multifocal disease, it is not clear to what extent molecular heterogeneity in these foci is a determining factor in the natural history of the disease or its role as a prognostic molecular biomarker for this disease.^32^, ^33^ The novelty of this work lies in the infrequency of molecular analyses of many foci in the same PCa with different lesion degrees (HGPIN, GL3, GL4, GL5, and LN). This approach allowed contributing to a better understanding of the molecular heterogeneity of a multifocal disease. In this study, five molecular subtypes associated with PCa were analyzed: *ERG*(+), *EZH2*, *NKX3.1*, *SPINK‐1*, and *SPOP* mutation, to establish their association with different lesion degrees and regional metastasis in LN in 20 PCa patients with poor prognosis, as well as the clonal origin of the foci in these patients. The *ERG*(+) subtype includes the *TMPRSS2‐ERG* fusion and *ERG* overexpression. Since no mutations were identified in the *SPOP* gene, which may be due to sample size, this was not considered for analysis.

Initially, when determining the presence of molecular subtypes, the *ERG*(+) subtype was found in 60% of the samples and in 85% of the patients. The previously reported frequency of this subtype for PCa in Caucasic men was between 40% and 66%,^3^, ^34^, ^35^, ^36^, ^37^ similar to what was found in the samples, but our frequency is higher in patients, probably because the present study includes HGPIN and LN, as well as various GL foci that previous studies did not analyze.

The reported *EZH2* subtype was identified in 54% of the samples and 85% of the patients, which was more frequently associated with the *ERG*(+) subtype (60% in samples and 82% in patients). All patients with *ERG*(−) (18%; *n =* 3) had the *EZH2* subtype. In the literature, the *EZH2*/*ERG*‐independent subtype has been reported in 30% of the cases by immunohistochemistry (IHC),^38^ which is similar to our results (40% in samples, with a decrease to 18% in patients). Nevertheless, it is important to note that our study analyzed many foci per patient, which, given the heterogeneity of PCa, increases the probability of finding an *ERG*(+) focus.

With respect to the *NKX3.1* subtype, 46% of the foci and 75% of the patients had this subtype, and the presence of *NKX3.1* subtype was more frequent in combination with *ERG*(+) (in 71% of the foci and 93% of the patients). Previous literature has not reported a frequency for this PCa subtype to compare our results. Given the relationship among the expressions of *ERG*, *EZH2*, and *NKX3.1*, these three subtypes (*ERG*(+)/*EZH2*/*NKX3.1*) were simultaneously present in 11 samples (13%) and in 11 patients (55%). It is important to highlight that these subtypes are also found separately, which indicates that the expression of these genes is more complex than the sole reciprocal relationship among them.^6^

The *SPINK‐1* subtype was found in 41% of the samples and 75% of the patients. Although most studies reported this subtype in *ERG*(−) PCa in approximately 10%–15% of the cases,^9^, ^15^, ^16^ *SPINK‐1* was present predominantly in combination with *ERG*(+) in our study (74% of *SPINK‐1* samples and 87% of the patients). Although *SPOP* mutation has been linked to *SPINK‐1* overexpression in various studies, mutations were not found in any foci.

Considering the simultaneous presence/absence of the four subtypes, more molecular patterns (15 patterns) were identified in the samples than in patients (8 patterns). It is noteworthy that the most frequent pattern in samples was an *ERG*(−) pattern: *EZH2*/*ERG*‐independent, which was found in 11 samples (13%); although this number is not too high, there were 3 more patterns that ranged between 10% and 11%. In patients, the most common pattern (40%) was the simultaneous presence of the four subtypes (*ERG*(+)/*EZH2*/*NKX3.1*/*SPINK‐1*), which is a relevant finding.

Similarly, a comparative analysis was carried out between clinicopathological characteristics, molecular subtypes, and BCR time in the 20 patients included in the study. No association was found between BCR time in patients, categorized as ≤3 and >3 months, and molecular subtypes or molecular concordance. Clinicopathological characteristics and the molecular subtype were compared with *ERG*, *EZH2*, *NKX3.1*, and *SPINK‐1* subtypes, which did not show any statistically significant association.

It is not surprising that the frequency of the analyzed subtypes increased when analyzed by patient (75%–85%) with respect to foci (41%–60%), while the presence of molecular patterns decreased. This is because most of the literature only analyzes one focus (index tumor)^23^ to establish subtypes, while the present study considers that a subtype is present when it is detected at least in one sample of a patient and includes a range of 3–5 samples (HGPIN, PCa foci, LN) from each patient. Molecular patterns decrease in patients because patterns with the presence of more subtypes increase when summing the presence of the subtypes in all samples analyzed by case.

The presence of molecular alteration of a subtype in at least one sample is significant because a clone with a specific subtype can be the most aggressive one and can determine prognosis and response to therapies. Thus, considering more adaptative tumoral cells, the methodology employed in this study to establish the presence of a subtype in LN is unusual but very significant and increases the presence of subtypes found in patients. The analyzed nonmolecular subtype was identified in 7 prostatic foci (8%); nevertheless, there are other molecular subtypes not evaluated in this work, such as mutations in the *IDH1* and *FOXA1* genes, described in the TCGA study.^3^ Interestingly, although the TCGA made a complete molecular analysis of PCa, 26% of the cases did not belong to any of the 7 subtypes described by them.

As for the concordance analysis of prostatic foci per patient, concordance among five molecular characteristics (*TMPRSS2‐ERG* fusion, expression of *ERG*, *EZH2*, *NKX3.1*, and *SPINK‐1*) was used to inquire about the clonal origin of PCa. When each molecular characteristic was evaluated separately, total concordance was the predominant state for most of them. Previous studies have found that 50%–60% of multifocal PCa tumors are concordant regarding the presence or absence of the *TMPRSS2‐ERG* fusion, similar to the 55% found in this study.^20^, ^34^, ^39^ There are no reports of concordance among foci regarding the expression of *ERG*, *NKX3.1*, or *EZH2*.

The second most frequent pattern for these four alterations was partially concordant cases (20%–45%). Among these, the molecular characteristics vary in relation to disease progression, as observed in subclonal alterations acquired during tumor cell evolution. Of the 5 molecular characteristics, 4 were alterations considered PCa subtypes that appear with disease progression (e.g., increased *ERG* expression that characterizes the *ERG*(+) subtype of late appearance). A progressive decrease in gene expression related to PCa was less frequent, only in *NKX3.1*. These results are not comparable with previous literature since other studies of several foci only analyzed 2 or 3 tumor samples without considering HGPIN and did not present results regarding Gleason score per focus.

Total molecular concordance, which includes concordant and partially concordant cases, was high (70%–90%) for each alteration and supports a monoclonal origin with subclonal events; between 15% and 30% of the cases show discordance, which could be associated with a multiclonal origin. When analyzing all alterations simultaneously per patient, the degree of discordance (55%) was higher than when alterations were evaluated separately (10%–30%), as expected. Other studies have reported a decrease in molecular concordance as the number of analyzed molecular alterations increases; in particular, the whole genome or transcriptome shows that the foci vary widely in the same patient.^40^, ^41^, ^42^ However, based on the results of our study, it cannot be discarded that some multifocal PCa may have a monoclonal and others a multiclonal origin; that is, there is no single origin for all PCa.

It is noteworthy that there were intratumoral differences in foci with the same GL in 3 out of 6 patients who had samples with more than one focus with the same GL. Nevertheless, concordance in the other 3 patients supports the probability of a monoclonal origin. Wei et al.^22^ showed that the correlation between different PCa foci in an individual is low. These findings highlight the importance of evaluating the role of multiclonality, which presents different alterations and subtypes in the same patient, complicating its application in translational oncology since alterations other than the index tumor may be determining metastases.^23^, ^43^

LN were not considered for concordance analysis, but it should be noted that none of the 13 LN studied was the same as the other foci analyzed, which underlines that malignant cells need other subclonal events to form metastatic foci in LN. This is in accordance with what was said above about subclonal alterations, which must provide additional advantages for such sophisticated processes as metastasis.^40^

Regarding the association between subtypes and histopathological groups, it was possible to observe all of them in HGPIN lesions, which leads to the conclusion that all the evaluated alterations are early events in the natural history of PCa. It is worth noting that the constant in all but one LN molecular subtype was the *ERG*(+) and *NKX3.1* subtype (92%). The molecular pattern with the four PCa subtypes (*ERG*[+]/*EZH2*/*NKX3.1*/*SPINK‐1*) was detected only in LN (23%). The 13 analyzed LN had more similarity among them than with the prostatic foci of the same patient. Findings in molecular patterns are in accordance with less heterogeneity in more advanced lesions (GL5 and LN). The result of less intermetastatic heterogeneity was expected when mutations are needed in the same genes to confer the same properties to a metastatic microenvironment at a distant site.

Only the expression of the *NKX3.1* gene was significantly different among the histopathological groups. Its expression decreased in more advanced lesions. Thus, the lowest levels were found in the LN group, indicating regional metastasis; this finding is consistent with other reports where *NKX3.1* has been found downregulated in many PCa tissues. It has been reported lost or completely eliminated even in the most dysplastic and metastatic PCa tumors.^11^, ^44^, ^45^, ^46^

No association was found between BCR time and clinicopathological characteristics, molecular subtypes, or molecular concordance, which may be due to our sample size and highlights the importance of future studies with larger sample sizes that are more representative.

## 5 CONCLUSIONS

The results of this study support the notion that, given the molecular heterogeneity of PCa, the molecular evaluation of a single focus does not provide a complete molecular picture of the disease in each patient. Although it is possible to assign molecular subtypes in this way, this type of analysis hinders the association of molecular subtypes with prognosis and the choice of appropriate treatment. Therefore, it would have a significant clinical application, but it is necessary to have results from several studies that manage to demonstrate the impact of these molecular subtypes on the prognosis and progression of the disease. All the subtypes examined here are of early onset, starting in preneoplastic lesions. *NKX3.1* expression level is the only molecular characteristic analyzed that shows a progression pattern.

## AUTHOR CONTRIBUTIONS

All authors have participated in the research that resulted in this work, as well as in the writing, reviewing, and final approval of this manuscript. Yenifer Yamile Segura‐Moreno and Martha Lucía Serrano wrote the main manuscript text and prepared the figures and tables.

## CONFLICT OF INTEREST

The authors declare no conflicts of interest.

## ETHICS STATEMENT

The present research was approved by the Ethics Committee of the Instituto Nacional de Cancerología, Bogotá, Colombia. Since tissues were obtained as part of routine care and not specifically for this study, and data used in this study were previously anonymized, the Ethics Committee of the Instituto Nacional de Cancerología (Bogotá, Colombia) waived the need for informed consent for this study. All methods complied with relevant guidelines and regulations at the national and international levels.

## Supporting information


**Appendix S1** Supplementary tables.

## Data Availability

All data generated or analysed during this study are included in this published article.
