# Supplementary material for: Determination of ERG(+), EZH2, NKX3.1, and SPINK‐1 subtypes to evaluate their association with clonal origin and disease progression in multifocal prostate cancer
Source: Cancer Rep (Hoboken). 2022 Oct 5;6(2):e1728. doi: 10.1002/cnr2.1728 (PMC9940006; doi:10.1002/cnr2.1728)
Supplement: Supplementary file 1 — Appendix S1 Supplementary tables. [file CNR2-6-e1728-s001.docx]

**Table 1S.** Clinicopathological and molecular characteristics of the 20 patients analyzed by presence of the ERG subtype

| **Characteristic** | **ERG Subtype** | | **p-value** |
| --- | --- | --- | --- |
|  | **Positive**  **(n = 14)** | **Negative**  **(n = 6)** |  |
| **Age of diagnosis (years)** |  |  |  |
| Median [IQR] | 66.5 [6.50] | 56.0 [15.2] | 0.432 |
| **Prediagnosis_PSA, ng/ml** |  |  |  |
| Median [IQR] | 15.0 [14.0] | 9.84 [8.33] | 0.629 |
| **Gleason score, n (%)** |  |  |  |
| 7 | 13 (68.4) | 6 (31.6) | 1.00 |
| 8 | 1 (100) | 0 (0.00) |  |
| **Gleason grade group, n (%)** |  |  |  |
| G2 | 3 (60.0) | 2 (40.0) | 1.00 |
| G3 | 10 (71.4) | 4 (28.6) |  |
| G4 | 1 (100) | 0 (0.00) |  |
| **Tumoral percentage (%)** |  |  |  |
| Mean ± SD | 39.6 ± 20.6 | 38.7 ± 17.3 | 0.926 |
| **Index diameter** |  |  |  |
| Mean ± SD | 2.04 ± 0.51 | 1.85 ± 0.41 | 0.443 |
| **Margin status in RP, n (%)** |  |  |  |
| Positive | 11 (68.8) | 5 (31.2) | 1.00 |
| Negative | 3 (75.0) | 1 (25.0) |  |
| **Perineural invasion, n (%)** |  |  |  |
| Intraprostatic | 5 (62.5) | 3 (37.5) | 0.642 |
| Intraprostatic + extraprostatic | 9 (75.0) | 3 (25.0) |  |
| **Lymphovascular invasion, n (%)** |  |  |  |
| Yes | 7 (70.0) | 3 (30.0) | 1.00 |
| No | 6 (66.7) | 3 (33.3) |  |
| Unknown | 1 (100) | 0 (0.00) |  |
| **Pathologic stage.pT, n (%)** |  |  |  |
| 2c-3a | 5 (71.5) | 2 (28.6) | 1.00 |
| 3b | 9 (69.2) | 4 (30.8) |  |
| **Pathologic stage.pN, n (%)** |  |  |  |
| 0 | 2 (50.0) | 2 (50.0) | 0.549 |
| 1 | 12 (75.0) | 4 (25.0) |  |
| **EZH2, n (%)** |  |  |  |
| Positive | 10 (66.7) | 5 (33.3) | 1.00 |
| Negative | 4 (80.0) | 1 (20.0) |  |
| **NKX3.1, n (%)** |  |  |  |
| Positive | 7 (77.8) | 2 (22.2) | 0.642 |
| Negative | 7 (63.6) | 4 (36.4) |  |
| **SPINK-1, n (%)** |  |  |  |
| Positive | 10 (83.3) | 2 (16.7) | 0.161 |
| Negative | 4 (50.0) | 4 (50.0) |  |
| **Molecular_concordance, n (%)** |  |  |  |
| Concordant | 1 (33.3) | 2 (66.7) | 0.216 |
| Partially concordant | 4 (66.7) | 2 (33.3) |  |
| Discordant | 9 (81.8) | 2 (18.2) |  |

IQR: Interquartile range; SD: Standard deviation, RP: Radical prostatectomy.

**Table 2S.** Clinicopathological and molecular characteristics of the 20 patients analyzed by presence of the EZH2 subtype

| **Characteristic** | **EZH2** | | **p-value** |
| --- | --- | --- | --- |
|  | **Positive**  **(n = 15)** | **Negative**  **(n = 5)** |  |
| **Age of diagnosis (years)** |  |  |  |
| Median [IQR] | 66.0 [16.5] | 66.0 [3.00] | 0.484 |
| **Prediagnosis_PSA, ng/ml** |  |  |  |
| Median [IQR] | 15.6 [12.9] | 8.00 [5.90] | 0.179 |
| **Gleason score, n (%)** |  |  |  |
| 7 | 15 (78.9) | 4 (21.1) | 0.250 |
| 8 | 0 (0.00) | 1 (100) |  |
| **Gleason grade group, n (%)** |  |  |  |
| G2 | 4 (80.0) | 1 (20.0) | 0.311 |
| G3 | 11 (78.6) | 3 (21.4) |  |
| G4 | 0 (0.00) | 1 (100) |  |
| **Tumoral percentage (%)** |  |  |  |
| Mean ± SD | 39.3 ± 18.8 | 39.4 ± 22.7 | 0.989 |
| **Index diameter** |  |  |  |
| Mean ± SD | 1.99 ± 0.46 | 1.94 ± 0.58 | 0.836 |
| **Margin status in RP, n (%)** |  |  |  |
| Positive | 12 (75.0) | 4 (25.0) | 1.00 |
| Negative | 3 (75.0) | 1 (25.0) |  |
| **Perineural invasion, n (%)** |  |  |  |
| Intraprostatic | 6 (75.0) | 2 (25.0) | 1.00 |
| Intraprostatic + extraprostatic | 9 (75.0) | 3 (25.0) |  |
| **Lymphovascular invasion, n (%)** |  |  |  |
| Yes | 8 (80.0) | 2 (20.0) | 1.00 |
| No | 7 (77.8) | 2 (22.2) |  |
| Unknown | 0 (0.00) | 1 (100) |  |
| **Pathologic_stage.pT, n (%)** |  |  |  |
| 2c-3a | 6 (85.7) | 1 (14.3) | 0.613 |
| 3b | 9 (69.2) | 4 (30.8) |  |
| **Pathologic_stage.pN, n (%)** |  |  |  |
| 0 | 3 (75.0) | 1 (25.0) | 1.00 |
| 1 | 12 (75.0) | 4 (25.0) |  |
| **ERG, n (%)** |  |  |  |
| Positive | 10 (71.4) | 4 (28.6) | 1.00 |
| Negative | 5 (83.3) | 1 (16.7) |  |
| **NKX3.1, n (%)** |  |  |  |
| Positive | 6 (66.7) | 3 (33.3) | 0.617 |
| Negative | 9 (81.8) | 2 (18.2) |  |
| **SPINK-1, n (%)** |  |  |  |
| Positive | 10 (83.3) | 2 (16.7) | 0.347 |
| Negative | 5 (62.5) | 3 (37.5) |  |
| **Molecular_concordance, n (%)** |  |  |  |
| Concordant | 2 (66.7) | 1 (33.3) | 1.00 |
| Partially concordant | 5 (83.3) | 1 (16.7) |  |
| Discordant | 8 (72.7) | 3 (27.3) |  |

IQR: Interquartile range; SD: Standard deviation; RP: Radical prostatectomy.

**Table 3S.** Clinicopathological and molecular characteristics of the 20 patients analyzed by presence of the NKX3.1 subtype

| **Characteristic** | **NKX3.1** | | **p-value** |
| --- | --- | --- | --- |
|  | **Positive**  **(n = 9)** | **Negative**  **(n = 11)** |  |
| **Age of diagnosis (years)** |  |  |  |
| Median [IQR] | 67.0 [3.00] | 60.0 [17.0] | 0.269 |
| **Prediagnosis_PSA, ng/ml** |  |  |  |
| Median [IQR] | 19.7 [11.9] | 11.5 [7.85] | 0.160 |
| **Gleason score, n (%)** |  |  |  |
| 7 | 8 (42.1) | 11 (57.9) | 0.450 |
| 8 | 1 (100) | 0 (0.00) |  |
| **Gleason grade group, n (%)** |  |  |  |
| G2 | 2 (40.0) | 3 (60.0) | 0.804 |
| G3 | 6 (42.8) | 8 (57.2) |  |
| G4 | 1 (100) | 0 (0.00) |  |
| **Tumoral percentage (%)** |  |  |  |
| Mean ± SD | 36.4 ± 20.7 | 41.6 ± 18.6 | 0.562 |
| **Index diameter** |  |  |  |
| Mean ± SD | 1.82 ± 0.52 | 2.11 ± 0.43 | 0.192 |
| **Margin status in RP, n (%)** |  |  |  |
| Positive | 6 (37.5) | 10 (62.5) | 0.284 |
| Negative | 3 (75.0) | 1 (25.0) |  |
| **Perineural invasion, n (%)** |  |  |  |
| Intraprostatic | 6 (75.0) | 2 (25.0) | 0.064 |
| Intraprostatic + extraprostatic | 3 (25.0) | 9 (75.0) |  |
| **Lymphovascular invasion, n (%)** |  |  |  |
| Yes | 4 (40.0) | 6 (60.0) | 1.00 |
| No | 4 (44.4) | 5 (55.6) |  |
| Unknown | 1 (100) | 0 (0.00) |  |
| **Node involvement, n (%)** |  |  |  |
| Yes | 6 (40.0) | 9 (60.0) | 0.617 |
| No | 3 (60.0) | 2 (40.0) |  |
| **Pathologic_stage.pT, n (%)** |  |  |  |
| 2c-3a | 3 (42.8) | 4 (57.2) | 1.00 |
| 3b | 6 (46.2) | 7 (53.8) |  |
| **Pathologic_stage.pN, n (%)** |  |  |  |
| 0 | 2 (50.0) | 2 (50.0) | 1.00 |
| 1 | 7 (43.7) | 9 (56.2) |  |
| **ERG, n (%)** |  |  |  |
| Positive | 7 (50.0) | 7 (50.0) | 0.642 |
| Negative | 2 (33.3) | 4 (66.7) |  |
| **EZH2, n (%)** |  |  |  |
| Positive | 6 (40.0) | 9 (60.0) | 0.617 |
| Negative | 3 (60.0) | 2 (40.0) |  |
| **SPINK-1, n (%)** |  |  |  |
| Positive | 4 (33.3) | 8 (66.7) | 0.362 |
| Negative | 5 (62.5) | 3 (37.5) |  |
| **Molecular_concordance, n (%)** |  |  |  |
| Concordant | 0 (0.00) | 3 (100) | 0.168 |
| Partially concordant | 2 (33.3) | 4 (66.7) |  |
| Discordant | 7 (63.6) | 4 (36.4) |  |

IQR: Interquartile range; SD: Standard deviation; RP: Radical prostatectomy.

**Table 4S.** Clinicopathological and molecular characteristics of the 20 patients analyzed by presence of the SPINK-1 subtype

| **Characteristic** | **SPINK-1** | | **p-value** |
| --- | --- | --- | --- |
|  | **Positive**  **(n = 12)** | **Negative**  **(n = 8)** |  |
| **Age of diagnosis (years)** |  |  |  |
| Median [IQR] | 63.0 [21.0] | 66.5 [4.25] | 0.486 |
| **Prediagnosis_PSA, ng/ml** |  |  |  |
| Median [IQR] | 8.25 [8.10] | 17.0 [10.6] | 0.160 |
| **Gleason score, n (%)** |  |  |  |
| 7 | 11 (57.8) | 8 (42.2) | 1.00 |
| 8 | 1 (100) | 0 (0.00) |  |
| **Gleason grade group, n (%)** |  |  |  |
| G2 | 3 (60.0) | 2 (40.0) | 1.00 |
| G3 | 8 (57.2) | 6 (42.8) |  |
| G4 | 1 (100) | 0 (0.00) |  |
| **Tumor percentage (%)** |  |  |  |
| Mean ± SD | 42.9 ± 20.5 | 33.9 ± 16.9 | 0.315 |
| **Index diameter** |  |  |  |
| Mean ± SD | 2.04 ± 0.52 | 1.89 ± 0.43 | 0.497 |
| **Margin status in RP, n (%)** |  |  |  |
| Positive | 9 (56.3) | 7 (43.7) | 0.618 |
| Negative | 3 (75.0) | 1 (25.0) |  |
| **Perineural invasion, n (%)** |  |  |  |
| Intraprostatic | 4 (50.0) | 4 (50.0) | 0.648 |
| Intraprostatic + extraprostatic | 8 (66.7) | 4 (33.3) |  |
| **Lymphovascular invasion, n (%)** | | | |
| Yes | 5 (50.0) | 5 (50.0) | 0.349 |
| No | 7 (77.8) | 2 (22.2) |  |
| Unknown | 0 (0.00) | 1 (100) |  |
| **Pathologic _stage.pT, n (%)** |  |  |  |
| 2c-3a | 6 (85.7) | 1 (14.3) | 0.157 |
| 3b | 6 (46.2) | 7 (53.8) |  |
| **Pathologic_stage.pN, n (%)** |  |  |  |
| 0 | 4 (100) | 0 (0.00) | 0.116 |
| 1 | 8 (50.0) | 8 (50.0) |  |
| **ERG, n (%)** |  |  |  |
| Positive | 10 (71.4) | 4 (28.6) | 0.161 |
| Negative | 2 (33.3) | 4 (66.7) |  |
| **EZH2, n (%)** |  |  |  |
| Positive | 10 (66.7) | 5 (33.3) | 0.347 |
| Negative | 2 (40.0) | 3 (60.0) |  |
| **NKX3.1, n (%)** |  |  |  |
| Positive | 4 (44.4) | 5 (55.6) | 0.361 |
| Negative | 8 (72.7) | 3 (27.3) |  |
| **Molecular_concordance, n (%)** |  |  |  |
| Concordant | 1 (33.3) | 2 (66.7) | 0.686 |
| Partially concordant | 4 (66.7) | 2 (33.3) |  |
| Discordant | 7 (63.6) | 4 (36.4) |  |

IQR: Interquartile range; SD: Standard deviation; RP: Radical prostatectomy.
